# Effectiveness of multipronged intervention on knowledge and attitude regarding health risk behaviors and prevention among Indian adolescents: A cluster randomized controlled trial

**DOI:** 10.1016/j.mex.2026.104181

**Published:** 2026-09-25

**Authors:** Manjula, Baby S Nayak, Shashidhara YN, Linu Sara George, Judith A Noronha, Ravindra Neelakanthappa Munoli, Veena G Kamath, Vasudeva Guddattu

**Affiliations:** a Department of Community Health Nursing, Manipal College of Nursing, Manipal Academy of Higher Education, Manipal, India; b Department of Paediatric Nursing, Manipal Academy of Higher Education, Manipal, India; c Department of Fundamentals of Nursing, Manipal College of Nursing, Manipal Academy of Higher Education, Manipal, India; d Department of Obstetrical and Gynecological Nursing, Manipal Academy of Higher Education, Manipal, India; e Department of Psychiatry, Kasturba Medical College, Manipal Academy of Higher Education, Manipal, India; f Department of Community Medicine, Kasturba Medical College, Manipal Academy of Higher Education, Manipal, India; g Department of Applied Statistics and Data Science, Prasanna School of Public Health, Manipal Academy of Higher Education, Manipal, India

**Keywords:** Adolescents, Multipronged approach, Health risk behavior, Knowledge, Attitude

## Abstract

Adolescence is a critical window for the emergence of lifestyle-related health risk behaviours that contribute substantially to the global burden of disease. This study examined the effectiveness of a multipronged, school-based intervention in improving knowledge and attitudes regarding health risk behaviours among adolescents.

A cluster-randomized design was used to recruit 338 first-year pre-university students (170 intervention; 168 control).Baseline data on sociodemographic characteristics, knowledge, and attitudes were collected using validated self-administered tools. The intervention comprised an interactive awareness session and roleplay focusing on causes, prevention, and management of health risk behaviours.

Participants were predominantly 16 years old and female across both groups. Substance use among family members was reported by 19.8% (intervention) and 18.2% (control). Significant improvements in knowledge were observed both within the intervention group over time [F(2336)=3.13, p < 0.004] and between the intervention and control groups [F(1336)=79.8, p < 0.001]. Attitude scores also showed significant positive changes over time within the intervention group [F(2336)=14.51, p < 0.001].

The multipronged intervention effectively enhanced adolescents’ knowledge and fostered more favourable attitudes towards health risk behaviour prevention. School based public health strategies that integrate education and skill building represent a practical, scalable approach for reducing multiple risk behaviours among adolescents.

## Graphical abstract

**Figure:**
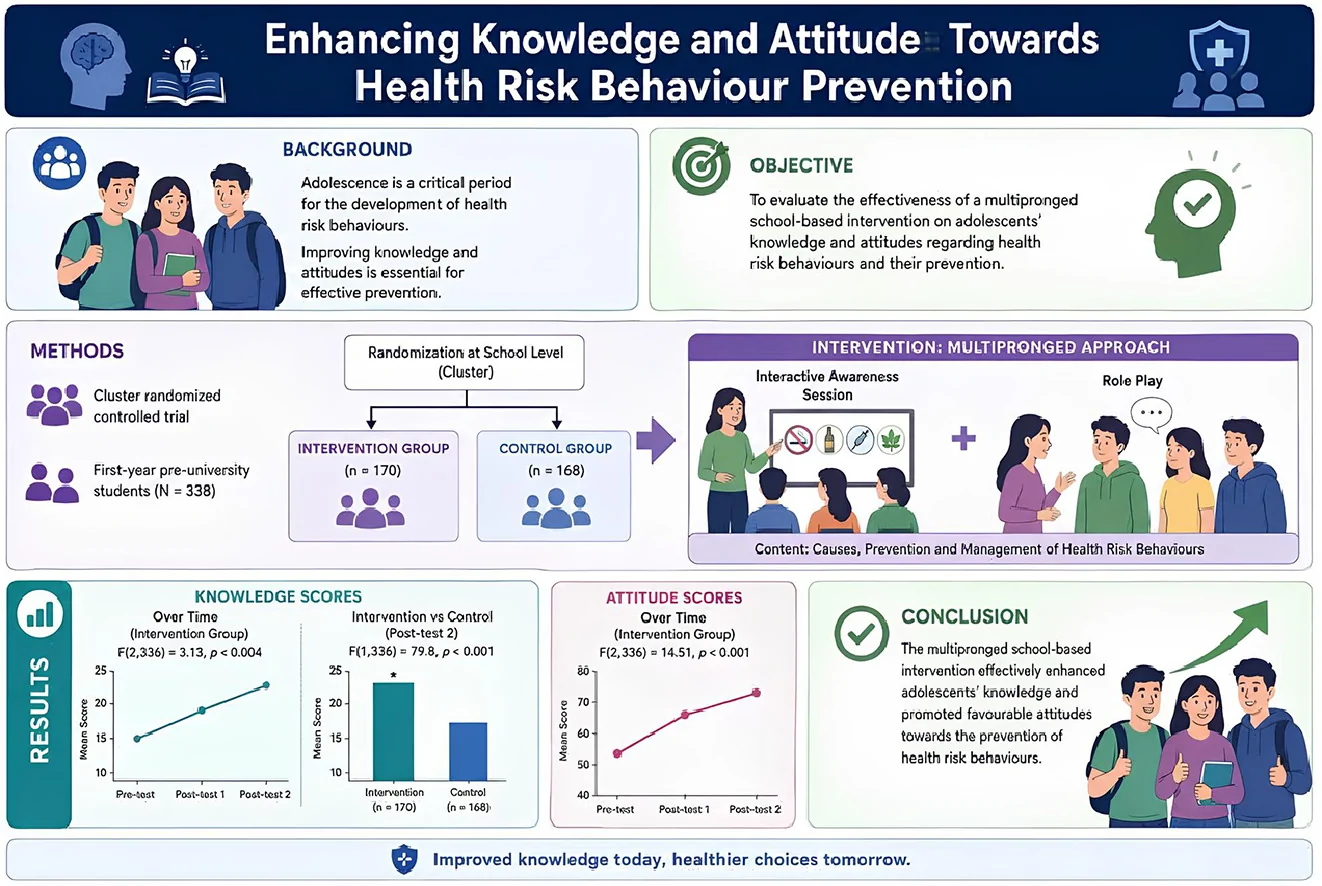

## Specifications table

**Table:** 

**Subject area**	Psychology
**More specific subject area**	Adolescent health
**Name of your method**	Cluster Randomized Control design
**Name and reference of original method**	Not applicable
**Resource availability**	Not applicable

## Background

Adolescents are increasingly exposed to health‑risk behaviours such as tobacco use, alcohol consumption, unhealthy dietary patterns, physical inactivity, and unsafe sexual practices. These behaviours, often formed during the critical developmental period of adolescence, substantially influence immediate well‑being and increase susceptibility to chronic diseases later in adulthood [1].

Global evidence indicates that unhealthy lifestyle patterns—including substance use, poor diet, and physical inactivity—remain major contributors to preventable morbidity and mortality among adolescents, with many of these risk behaviours beginning early and persisting into adulthood [2]. Research findings further highlight that multiple risk behaviours tend to cluster and track over time, exacerbating long‑term health risks [3].

Despite the presence of health education programs, adolescents frequently lack adequate knowledge and hold permissive attitudes toward these risk behaviours, reducing the effectiveness of prevention strategies aimed at promoting healthy lifestyle choices [4].

Adolescence is a pivotal period marked by significant physical, cognitive, and social development, during which behaviours that are both health-promoting and risky are established and often carried into adulthood. Studies show that many non-communicable diseases trace their roots to health risk behaviours initiated during adolescence, such as tobacco and alcohol use, poor diet, physical inactivity, unsafe sexual practices, and occasional substance abuse. Globally, over 50% of the disease burden linked to non-communicable diseases is attributed to health-risk behaviours often adopted in youth [5].

A range of preventive interventions, particularly school‑based and peer‑led programs, have been implemented and evaluated in response to these concerns.

A scientific report of a systematic review and meta-analysis of whole‑school interventions found significant reductions in cyber‑bullying (25%), regular smoking (31%), and cyber‑aggression (37%) among adolescents. However, the review reported no statistically significant changes in alcohol use, recreational drug use, or mental health outcomes, which the authors attribute to variability in implementation fidelity and challenges in adapting interventions to diverse school contexts [6].

The school-based interventions demonstrated notable effectiveness in promoting behavioural change and reducing sexual risk-taking among adolescents. This was primarily achieved through educational initiatives that raised awareness of risky sexual behaviours, addressed adolescent reproductive health issues, and enhanced knowledge of the adverse outcomes associated with such behaviours [7].

Region-specific evaluations highlight further insights. A cluster-randomized controlled trial in Chandigarh, India, demonstrated that a school-based behavioural change intervention significantly reduced key chronic disease risk factors among adolescents, underscoring the feasibility of localized school-centred strategies [8]. Concurrently, a scoping review spanning 2009–2023 across diverse adolescent populations found that nine peer-reviewed intervention programs yielded mild to moderate success, particularly those combining school-based counselling, electronic screening, and personalized feedback, whereas incentive-based methods showed negligible efficacy [5].

Furthermore, technological innovations such as web-based interventions have been shown to positively influence health behaviour modification. A systematic review (2016–2021) reported that 13 out of 14 such interventions achieved significant improvements in adolescents’ diet, physical activity, and substance use behaviours, although the overall quality of evidence was rated low due to small sample sizes and short follow-up durations [9].

School-based interventions through peer education, web platforms, or whole-school approaches frequently involve multiple components: knowledge dissemination, attitude reshaping, skill development, electronic screening, counselling, and tailored feedback [10,11]. Interventions consistently produced moderate to large gains in health-related knowledge and attitudes; however, behavioural change outcomes varied, particularly for substance use, road safety and sexual risk behaviours. Variations in intervention fidelity, theoretical underpinnings, cultural adaptation, and participatory design processes limit consistency and replication across settings [12,13].

## Method details

### Population

Eligible participants included adolescents aged between 16 and 18 years, enrolled in Science, Arts, or Commerce streams, from both urban and rural settings, available during the data collection period and provided informed consent.

### Procedures

The study implemented a multistage cluster sampling technique. Pre-university colleges and adolescents were selected from seven taluks in the Udupi district of Karnataka, India. Out of these colleges, based on the required sample size, a total of six pre-university colleges were randomly chosen by the lottery method. Within these institutions, clusters of adolescents who met the inclusion criteria were allocated into experimental and control groups. Calculated sample size for each arm was 167; considering the design effect of 2, the total sample size calculated for intervention and control arm was 334. The number of colleges were calculated based on the total sample size, design effect of 2 & the average intake of 60 students per cluster.

Based on the calculation, the number of colleges included in the study was 6. Three colleges were randomly selected for the intervention and the control arm, respectively. Since it’s a cluster design, the final sample size included for the study was 342(172 in the intervention and 170 in the control arm). The sample size included for the final analysis was 338(170 in the intervention and 168 in the control arm) excluding the sample dropouts of two each from the intervention and control arm, respectively.

### Procedure for data collection

The data were collected using the structured knowledge questionnaire, which consists of 32 items on safety behavior while driving, violence, bullying, suicidal ideation, smoking, tobacco, alcohol, drugs and other inhalant use, problems associated with eating, physical inactivity, and sexual problems.

A structured attitude questionnaire consisting of 36 items on safety behaviour while driving, violence, bullying, suicidal ideation, smoking, tobacco, alcohol, drugs and other inhalant use, problems associated with eating, physical inactivity, and sexual problems was used to collect data on adolescents’ attitude towards risk behaviour.

Data collection for the pretest, implementation of the multipronged approach, and the posttest I after one month and post-test II at three months were carried out between June and December 2024. Detailed information about the study was provided to the participants on the first day through a participant information sheet. Informed consent forms were distributed to parents through class teachers, and assent was obtained from adolescents whose parents provided written consent. On the following day, the demographic questionnaire, structured knowledge questionnaire and a questionnaire to assess the adolescents’ attitude on risk behaviors were administered.

On the first visit, after briefing on the participant information sheet, informed consent and assent forms were given to the adolescents; during the second visit, after collecting the consent forms, a pretest was conducted on knowledge and attitude on health risk behaviour. On the third visit, a role play on health risk behaviours, it’s causes, consequences were held including briefing and summarization of role play, demonstration of YOGA and guided imagery relaxation technique by the expert. During the fourth visit, for the adolescents, an awareness session on prevention and management of risk behaviour and for the teachers, an awareness session on identification of risk behaviour among adolescents, and the process of referral was held by the psychiatrist.

A follow up visit to conduct post-test I and the reinforcement sessions were carried out after a month. Leaflets briefing on the health risk behaviour, causes, consequences and management were issued. The adolescent clubs were initiated in the colleges with the principal as the chairperson, teacher coordinator and two student representatives from each class. The second follow-up visit was carried out to conduct post-test II, and reinforcement sessions were carried out. After the completion of interventions for the intervention group, awareness sessions for the control groups were organized, the leaflets briefing on the health risk behaviour, causes, consequences and management were issued.

### Statistical analysis

IBM Statistical Package for Social Sciences (SPSS) version 20.0 and Jamovi 2.3 software was used for data analysis. Frequency and percentages were computed to describe the sample characteristics. Mean, frequency and percentage, standard deviation, minimum and maximum scores were calculated to describe the knowledge and attitude in the intervention and control groups. Repeated-measures ANOVA (RM ANOVA) was used to determine the effectiveness of the multipronged approach on knowledge and attitude regarding health risk behavior among adolescents.

**Section 1: Description of sample characteristics**

The demographic characteristics are presented as frequencies and percentages, revealing that a total of 73.3% of the participants in the intervention group and 90% in the control group were 16 years old a-; 52.9% in the intervention group and 52.4% in the control group were females; Substance use among family members was reported as 19.8% and 18.2% in the intervention and control groups, respectively.

The data presented in Table 1 show the pre- and posttest knowledge scores in the intervention and control groups. The maximum obtained scores were similar at baseline. The mean scores were found to be increased in the intervention group from pretest to posttest I, ranging from 12.2 (SD=4.14) to 14.2 (4.28) and returning to baseline scores with a mean of 12.2 (SD=4.29), whereas in the control group, they were found to decrease from baseline to posttest I 10.8 (SD=9.54) and slightly increase to 10.0 (SD=4.41). The intervention group had higher mean scores than the control group.

**Table 1:** Pretest and posttest mean, standard deviation, minimum and maximum knowledge scores in the intervention and control groups.

Knowledge score	Intervention arm				Control arm			
	Min	Max	Mean	SD	Min	Max	Mean	SD
Baseline(Pretest)	3	22	12.2	4.14	0	20	10.8	4.70
Posttest I	4	25	14.2	4.28	0	20	9.54	3.93
Post -test II	2	21	12.2	4.29	2	20	10.0	4.41

*Note:* N=(170+168); Min-Minimum obtained score; Max-Maximum obtained score; SD-Standard deviation.

**Section 2: Frequency and Percentage Distribution of Knowledge Level on Health Risk Behavior in the Intervention and Control Group**

The knowledge level was categorized as poor (0–10), average (11–21) and good (22–32). The frequency and percentage of knowledge scores of the intervention and control groups are presented in Table 2.

**Table 2:** Frequency and percentage distribution of knowledge level on health risk behavior in the intervention and control group.

Knowledge level	Intervention			Control		
	Pretest f (%)	Post test I f (%)	Post test II f (%)	Pretest f (%)	Post test I f (%)	Post test II f (%)
Good	—	3(0.3)	…	…	….	…
Average	92(9.1)	125(12.3)	94(9.3)	74(7.3)	54(5.3)	58(5.7)
Poor	78(7.7)	43(4.2)	75(7.4)	94(9.3)	114(11.2)	111(10.9)

*Note:* N = 170+168. f=frequency; % =percentage.

The data presented in Table 2 show that the majority (92, 9.1%) of the intervention participants had average knowledge of health risk behavior, and 94 (9.3%) of the control group had poor knowledge in the pretest. In posttest I, it was 125 (12.3%) in the intervention group and 114 (11.2%) in the control group.

**Section 3: Effectiveness of multipronged approach on knowledge on health risk behavior among adolescents**

RM ANOVA was used to determine the effectiveness of the multipronged approach on knowledge on health risk behavior among adolescents. Table 3.

**Table 3:** Repeated-measures ANOVA on knowledge scores between the intervention and control groups.

Knowledge	Mean square	F value	df	p	η²p
Time (Pre vs Posttest)	48.8	3.13	2, 336	0.004	0.009
Group (Intervention vs Control)	1935.4	79.8	1, 336	< .001	0.192
Time x Group	251.1	16.11	2, 336	< .001	0.046

Note: N = 338 (170 + 168) Time- within group effects; Group-between group effects; Time x Group-interaction effects; F-ratio; df=degree of freedom; p-level of significance; η²p = Partial Eta Squared (effect size).

The results of the repeated-measures ANOVA on the effectiveness of multipronged intervention on knowledge of adolescents on health risk behavior, which reveals statistical significance within the intervention group [F(2336)=3.13 p < 0.004 η²p = 0.009], between the intervention and control groups [F(1336)=79.8 p<.001 η²p = 0.192] and the interaction effect (Time x group) [F(2336)=16.11 p<.001 η²p = 0.046], indicating that the intervention group changed significantly over time in terms of improvement in knowledge on health risk behavior among adolescents.

The effect size within the group, between the group and between the time x group suggest that the change observed over time significantly differed within the intervention group and between the groups, which was due to the effect of the intervention. Compared with those in the control group, the knowledge level in the intervention group significantly improved.

**Section 4: Description of the attitude on health risk behavior of adolescents**

The data were collected using the Structured Attitude Questionnaire, which consists of 36 items on safety behavior while driving, violence, bullying, suicidal ideation, smoking, tobacco, alcohol, drugs and other inhalant use, problems associated with eating, physical inactivity, and sexual problems. The mean pretest and posttest scores at baseline at one month and at three months are presented in Table 4.

**Table 4:** Pretest and posttest mean, standard deviation, minimum and maximum attitude scores in the intervention and control groups.

Attitude	Intervention arm				Control arm			
	Min	Max	Mean	SD	Min	Max	Mean	SD
Baseline	43	152	113	18.2	38	147	114	20.3
Posttest I	61	155	116	15.9	41	157	120	20.7
Post -test II	63	158	115	14.4	41	147	109	14.9

*Note:* N=(170+168); Min-Minimum obtained score; Max-Maximum obtained score; SD-Standard deviation.

The data presented in Table 4 show the pre- and posttest attitude scores in the intervention and control group. The mean scores increased in the intervention group from pretest to posttest I, ranging from 113 (SD=18.2) to 116 (SD=15.9) and 115 (SD=14.4). In contrast, in the control group, alcohol consumption increased from baseline 114 (20.3) to posttest I 120 (SD=20.7) and slightly decreased to 109 (SD=14.9) in posttest II. Both the intervention and control groups presented with higher mean scores.

**Section 5: Frequency and Percentage Distribution of Attitude on Health Risk Behavior in the Intervention and Control Group**

The attitude was categorized as unfavorable (< 90) and favorable (>90–180). The frequency and percentage knowledge scores of the intervention and control groups are presented in Table 5

**Table 5:** Frequency and percentage distribution of attitude on health risk behavior in the intervention and control groups.

Attitude on health risk behavior	Intervention			Control		
	Pretest f (%)	Post test I f (%)	Post test II f (%)	Pretest f (%)	Post test I f (%)	Post test II f (%)
Unfavorable	13(1.3%)	8(0.8%)	8(1.8%)	18(1.8%)	12(1.2%	17(1.7%)
Favorable	157(15.5%)	163(16.1%)	161(15.9%)	150(14.8%)	156(15.4%)	151(14.9%)

*Note:* N = 170+168.

The data presented in the Table 5 show that there was an improvement in attitude score from baseline (pre-test) to posttest I & II, which was held at one- and three-month intervals both in the intervention and control group; from baseline 157(15.5%) to 163(16.1%) in the first follow-up after one month in the intervention group. In the second follow-up, after three months, the knowledge score was reduced slightly compared to the second follow-up, 161(15.9%), and remains higher than the baseline. Hence, it is inferred that the intervention was effective in improving the attitude towards health risk behavior and its prevention.

**Section 6: Effectiveness of multipronged approach e on health risk behavior among adolescents**

RM ANOVA was used to determine the effectiveness of the multipronged approach on knowledge on health risk behavior among adolescents.

The data in Table 6 provide the results of the repeated-measures ANOVA on the effectiveness of multipronged intervention on the attitude of adolescents regarding health risk behavior, which reveals statistical significance within the intervention group [F (2336) =14.51 p < 0.001 η²p = 0.041], indicating that the intervention group changed significantly over time in terms of favorable attitude. The effect size within the group, between the group and between the time x group suggests that there are no changes over time that differ within the intervention group and between the groups.

**Table 6:** Repeated-measures ANOVA on attitude scores between the intervention and control group.

Attitude	Mean square	F value	df	p	η²p
Time (Pre vs Posttest)	3232	14.51	2, 336	< .001	0.041
Group (Intervention vs Control)	118	0.244	1, 336	0.622	0.001
Time x Group	1905	8.56	2, 336	< .001	0.025

*Note*: N = 338 (170 + 168) Time- within group effects; Group-between group effects; Time x Group-interaction effects;.

F-ratio; df=degree of freedom; p-level of significance; η²p = Partial Eta Squared (effect size).

### Method validation

The findings of the present study are supported by a study conducted by Savitha [14], who similarly demonstrated the effectiveness of structured educational interventions in improving adolescents’ knowledge and attitudes regarding risk-taking behaviours. Varghese et al. [15]reported that the intervention produced a substantial improvement in adolescents’ knowledge of risk-taking behaviour, with scores increasing from minimal understanding at pre-test to adequate knowledge post-test. This confirms the effectiveness of structured educational programmes in enhancing awareness.

Although attitudes showed little statistical change, the emergence of more positive attitudes suggests early perceptual shifts that may strengthen with continued reinforcement. A study conducted by Taghdis et al. [16] reported that a structured school-based intervention significantly enhanced students’ attitudes, subjective norms, perceived behavioural control, and behavioural intentions towards fruit and vegetable consumption, with no baseline differences between groups. These post-intervention changes indicate that targeted educational strategies can meaningfully influence the cognitive and motivational factors that shape adolescent behaviour. The improvement in behavioural intention observed in their study supports the broader premise that strengthening knowledge and fostering positive attitudes can translate into healthier behavioural choices.

The four-week instructor-led intervention, knowledge improved across all HRB domains, with over 90% of participants achieving good knowledge levels. This consistent post-intervention gain underscores the effectiveness of targeted health education in enhancing adolescents’ understanding of risk behaviours and promoting healthier attitudes. These results highlight the value of integrating structured, evidence-based health education into school curricula as a viable strategy to mitigate HRBs and improve overall adolescent health outcomes [17,18].

Previous evidence supports the effectiveness of targeted health interventions in improving adolescents’ understanding of risky behaviours. A study conducted by Gupta et al. [19] observed that although all adolescents used the internet and social media, a substantial proportion (81%) showed psychological dependence. The intervention in that study produced a significant improvement in knowledge and a reduction in dependence scores (P < 0.001), with knowledge levels influenced by age and educational status. These findings align with the present study, highlighting that structured, developmentally appropriate interventions can successfully enhance knowledge and promote healthier attitudes among adolescents toward reducing risk behaviors.

The findings of the present study are supported by earlier evidence on the effectiveness of peer‑education interventions in modifying adolescents’ risk‑related knowledge and attitudes. A substantial rise in post‑test knowledge (21.32) and attitude scores (23.24) compared with pre‑test scores (12.91 and 14.61), with a highly significant t value of 27.17. This demonstrates that peer‑led education produces meaningful gains in awareness and positive attitudinal shifts. Similar improvements in the study reinforce that peer education provides an engaging and relatable platform for adolescents, thereby enhancing understanding of high‑risk behaviours and promoting healthier attitudes [20]. A Theory of Planned Behaviour(TPB) based intervention has been shown to significantly improve adolescents’ attitudes, perceived behavioural control, and intentions related to reducing risky internet use while also lowering internet addiction scores and enhancing academic performance [21].

Peer-led education (PLE) significantly improved adolescents' knowledge and attitude toward road safety. The mean knowledge score increased from 10.5 to 17.5, demonstrating a statistically significant improvement (*P* < 0.001). The mean attitude score also showed a positive increase from 46.7 to 48.1 following the intervention. A significant positive correlation was observed between knowledge and attitude (*P* = 0.04), indicating that improved knowledge was associated with a more favourable attitude toward road safety. Additionally, selected sociodemographic variables were significantly associated with knowledge and attitude outcomes [22].

The findings of the present study align with existing evidence emphasizing that empowering adolescents with accurate knowledge and practical skills enhances their ability to make informed decisions regarding their health and behaviour.

The empowerment equips young people with the capacity to understand risks, evaluate consequences, and take greater control over choices affecting their well‑being. This supports the observed improvement in knowledge and attitude following the intervention, suggesting that structured educational programs play a crucial role in strengthening adolescents’ decision‑making abilities and reducing engagement in risk behaviours. By fostering awareness and confidence, such interventions contribute to healthier behavioural choices and promote long‑term protective outcomes among adolescents [23].

The study findings of Deshpande [24]reinforce the effectiveness of structured educational interventions in improving adolescents’ knowledge and attitudes related to risk behaviours. A significant increase in understanding of sexuality issues and gender sensitivity (t = 20.85, p < 0.001) highlights the value of comprehensive, school-based education in addressing misconceptions and promoting healthier perspectives. Overall, these improvements across cognitive and emotional domains support the effectiveness of the intervention in fostering informed attitudes and reducing vulnerability to risk behaviours among adolescents. The study findings of Schilder [13] also demonstrated that targeted educational interventions can significantly enhance adolescents’ awareness of risky behaviours. The intervention group showed notably higher online risk awareness than the control group even after adjusting for gender and grade, indicating a substantial intervention effect. These findings align with the present study, further supporting that structured, skill‑building programs effectively improve adolescents’ knowledge and attitudes toward reducing risk behaviours [25].

The community‑based educational interventions that significantly enhanced adolescents’ knowledge and attitudes toward preventing risk behaviours; demonstrated substantial post‑intervention gains, with most adolescents achieving adequate knowledge (73.3%) and favourable attitudes (76.7%) regarding substance‑related health risks, confirming the effectiveness of structured educational strategies in influencing adolescent health behaviours [24].

Digital interventions have been found to have positive effects on knowledge and attitudes, but it is more difficult to modify behaviours with strictly digital interventions or combined with complementary face-to-face sessions or group class activities [26]. The intervention package implemented among adolescents by involving parents and teachers is an effective model for school-based behaviour-change interventions [8].

This study provides novel evidence on the effectiveness of a multipronged, school-based intervention combining interactive awareness sessions, role-play, YOGA and guided imagery to address multiple adolescent health risk behaviors. Using a rigorous cluster randomized design, the intervention significantly improved adolescents’ knowledge and attitudes toward health risk behavior prevention.

The findings highlight the value of participatory and skill-based learning approaches in promoting healthy decision-making during a critical developmental period. As a low-cost and scalable school-based strategy, the intervention has strong potential for replication across diverse settings and supports key public health priorities. Furthermore, the study contributes to the achievement of Sustainable Development Goals (SDGs) 3 (Good Health and Well-being), 4 (Quality Education), and 5 (Gender Equality) by promoting health literacy, empowering adolescents, and fostering equitable access to preventive health education.

### Limitations

The multipronged intervention reinforcement sessions were conducted only at baseline, the first and third months, and the study did not include a long-term follow-up assessment. Consequently, the long-term sustainability of the intervention effects could not be evaluated.

## Ethics statements

Administrative permission was obtained from the Deputy Director of the Pre-University Board and the heads of the Pre-University Colleges. The approval was obtained from the Institutional Ethics Committee, and the study was registered under the Clinical Trial Registry of India. Informed consent from the parents and assent from the study participants were obtained after the participant information sheet was explained. To ensure anonymity, the questionnaires were administered without any identification information from the participants.

## CRediT author statement

**Manjula:** Conceptualization, Methodology, Investigation, Data analysis, Original draft preparation; **Baby S Nayak:** Conceptualization, Methodology, Data analysis, Review and editing; **Shashidhara YN:** Conceptualization Methodology, Reviewing and editing; **Linu Sara George:** Review and editing; **Judith A Noronha:** Review and editing; **Veena G Kamath:** Review and editing; **Ravindra Neelakanthappa Munoli:** review and editing; **Vasudeva Guddattu:** Data analysis, Review and editing.

## Related research article

None.

## Declaration of competing interest

The authors declare that they have no known competing financial interests or personal relationships that could have appeared to influence the work reported in this paper.

## Data Availability

Data will be made available on request.
